# A novel recurrent mutation in *ATP1A3* causes CAPOS syndrome

**DOI:** 10.1186/1750-1172-9-15

**Published:** 2014-01-28

**Authors:** Michelle K Demos, Clara DM van Karnebeek, Colin JD Ross, Shelin Adam, Yaoqing Shen, Shing Hei Zhan, Casper Shyr, Gabriella Horvath, Mohnish Suri, Alan Fryer, Steven JM Jones, Jan M Friedman

**Affiliations:** 1 Division of Neurology, Department of Pediatrics, University of British Columbia and BC Children’s Hospital, Vancouver, BC V6H 3N1, Canada; 2 Division of Biochemical Diseases, Department of Pediatrics, University of British Columbia and BC Children’s Hospital, Vancouver, BC V6H 3N1, Canada; 3 Division of Translational Therapeutics, Department of Pediatrics, University of British Columbia and BC Children’s Hospital, Vancouver, BC V6H 3N1, Canada; 4 The Pharmaceutical Outcomes Programme, University of British Columbia, Vancouver, BC V5Z 4H4, Canada; 5 Department of Medical Genetics, University of British Columbia, Vancouver, BC V5Z 4H4, Canada; 6 Centre for Molecular Medicine and Therapeutics, Vancouver, BC V5Z 4H4, Canada; 7 Child & Family Research Institute, Vancouver, BC V5Z 4H4, Canada; 8 Canada's Michael Smith Genome Sciences Centre, British Columbia Cancer Agency, Vancouver, BC V5Z 4S6, Canada; 9 Department of Clinical Genetics, Nottingham University Hospitals National Health Service Trust, Nottingham NG5 1PB, UK; 10 Department of Clinical Genetics, Royal Liverpool Children’s Hospital, Liverpool L12 2AP, UK; 11 Department of Molecular Biology and Biochemistry, Simon Fraser University, Burnaby, BC V5A 1S6, Canada; 12 Medical Genetics Research Unit, Child & Family Research Insitute, Box 153, 4500 Oak Street, Vancouver, BC V6H 3N1, Canada

**Keywords:** CAPOS syndrome, Cerebellar ataxia, Optic atrophy, Sensorineural hearing loss, *ATP1A3*

## Abstract

**Background:**

We undertook genetic analysis of three affected families to identify the cause of dominantly-inherited CAPOS (cerebellar ataxia, areflexia, pes cavus, optic atrophy and sensorineural hearing loss) syndrome.

**Methods:**

We used whole-exome sequencing to analyze two families affected with CAPOS syndrome, including the original family reported in 1996, and Sanger sequencing to assess familial segregation of rare variants identified in the probands and in a third, apparently unrelated family with CAPOS syndrome.

**Results:**

We found an identical heterozygous missense mutation, c.2452G > A (p.(Glu818Lys)), in the Na^+^/K^+^ ATPase α_3_*(ATP1A3*) gene in the proband and his affected sister and mother, but not in either unaffected maternal grandparent, in the first family. The same mutation was also identified in the proband and three other affected members of the second family and in all three affected members of the third family. This mutation was not found in more than 3600 chromosomes from unaffected individuals.

**Conclusion:**

Other mutations in *ATP1A3* have previously been demonstrated to cause rapid-onset dystonia-parkinsonism (also called dystonia-12) or alternating hemiplegia of childhood. This study shows that an allelic mutation in *ATP1A3* produces CAPOS syndrome.

## Background

Cerebellar ataxia, areflexia, pes cavus, optic atrophy, and sensorineural hearing loss (CAPOS) syndrome (OMIM 601338) is a rare neurological disorder, which to date has only been reported in a single family. In 1996, Nicolaides et al. [1] described CAPOS syndrome in a brother and sister and their mother, all of whom were normal until they presented with a relapsing and partially remitting, early-onset cerebellar ataxia following a febrile illness. Other features included progressive optic atrophy and sensorineural hearing loss, generalized hypotonia, areflexia and pes cavus without evidence of a peripheral neuropathy on neurophysiological studies. All three patients shared these key features, although the severity and number of ataxic relapses varied. The mode of inheritance was thought to be autosomal dominant or mitochondrial. Extensive investigations failed to identify a cause, and the authors believed that the neurological disorder affecting this family probably represented a “new” “ataxia plus” syndrome. No other patients with CAPOS syndrome have been reported in the subsequent 17 years.

We have identified two additional families with CAPOS syndrome and reassessed the original family. We here describe the clinical features and natural history of this disorder and report a novel heterozygous missense mutation of the *ATP1A3* gene that causes CAPOS syndrome in all ten affected members of these three apparently unrelated families.

## Methods

We obtained informed consent and assent, when appropriate, from participating family members. Ethical review and approval according to the Finding Of Rare disease GEnes (FORGE) Canada Consortium and University of British Columbia were also obtained. Clinical details of Family 1, originally described in 1996 [1], were updated by A.F. in 2013.

**Figure 1:**
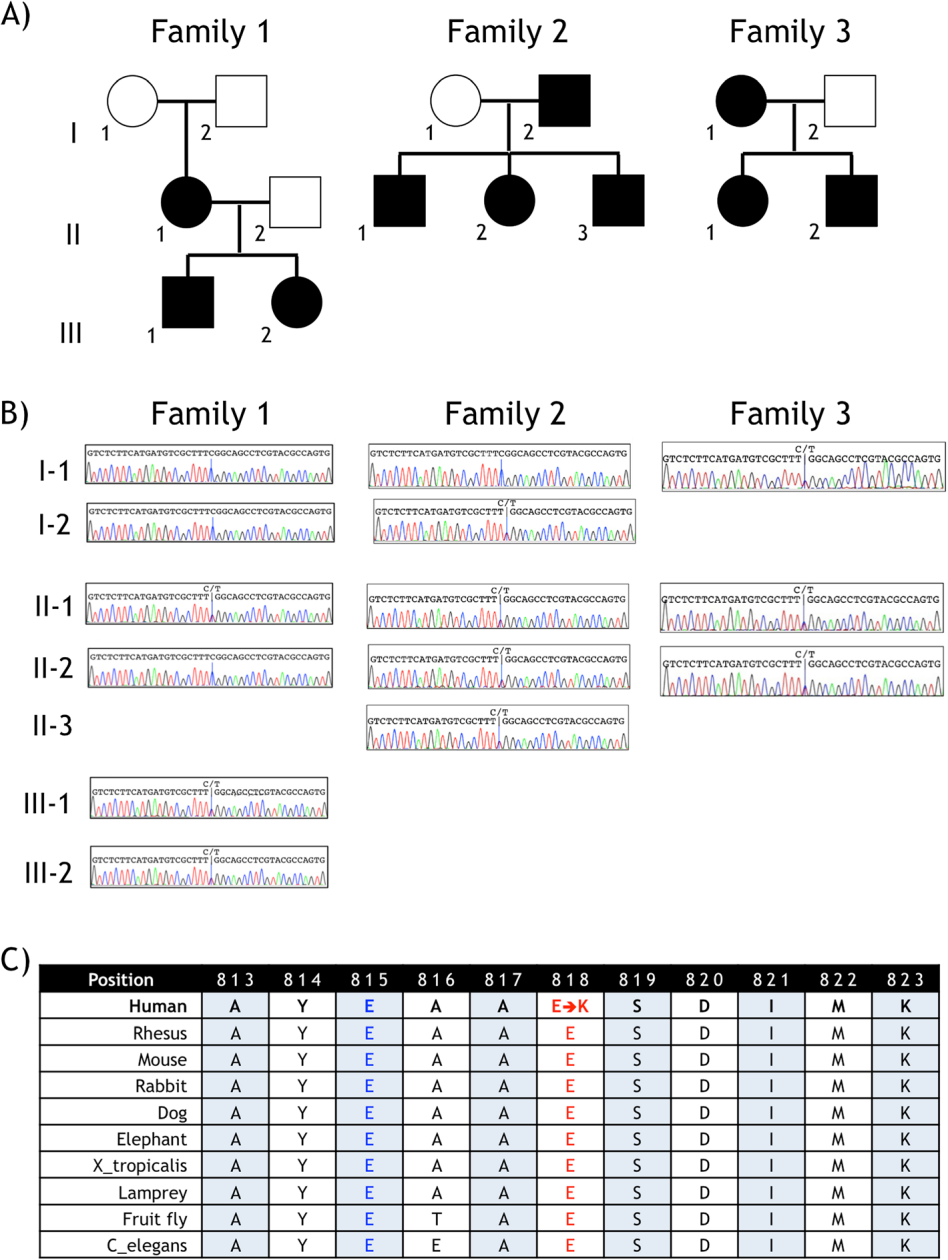
**Pedigrees and Sanger sequencing results in three families with CAPOS syndrome. A)** Pedigees. Family 1 was initially described by Nicolaides et al. [1] Individuals with CAPOS syndrome are indicated by filled pedigree symbols, and unaffected individuals, by empty symbols. **B)** Sanger sequencing results in affected and unaffected family members. A portion of the Sanger sequencing trace is shown for each individual who was tested, with the nucleotides at Ch19:47,166,267, corresponding to *ATP1A3* position c.2452 on the minus strand, indicated by a vertical blue line. Each affected individual is heterozygous for the variant T (corresponding to c.2452A) and the reference C (corresponding to c.2452G) nucleotides. **C)** Conservation of Na^+^/K^+^ ATPase α_3_ protein sequence in the region of the mutation. The E818K mutation found in all three CAPOS families is shown in red type; the location of the E815K loss-of-function mutation, which is associated with alternating hemiplegia of childhood, is shown in blue type. This segment of the Na^+^/K^+^ ATPase α_3_ protein is highly conserved.

We performed whole exome sequencing on DNA from the probands in Families 1 (Figure 1A, Family 1 III-1) and 2 (Figure 1A, Family 2 II-2) on an Illumina HiSeq 2000 after SureSelect Target enrichment with an Agilent 50 Mb Human All Exon Kit (Agilent Technologies Inc., USA) using the manufacturers’ protocols and other methods previously described [2]. Illumina's GA Pipeline was used to remove sequencing reads that failed chastity filtering, and the remaining reads were mapped to the human genome reference sequence (HG18) using BWA [3]. Duplicate reads and reads with a mapping score of 0 were removed, and the remaining aligned reads were exported to pileup format. Variants were identified using SAMtools [4]. We filtered out variants with a quality score below 10 at varFilter parameter − D 1000 for single nucleotide variants and varFilter parameters − D 1000, −d 2 and − l 30 for indels. We annotated the filtered variants as “known” or “novel”, depending on whether they had been previously reported in dbSNP (version 129 or 130) [5,6] or in the 1000 Genomes Project [7,8]. We also determined how many times the variant had previously been observed in our in-house database of 1834 normal germline genomes sequenced at Canada’s Michael Smith Genome Sciences Centre.

## Results

Table 1 provides an overview of the clinical features of these three, apparently unrelated families. Additional file 1: Table S1 summarizes the results of investigations performed in the families.

**Table 1:** Clinical features of 10 patients from three families with CAPOS syndrome

	**Family 1 (Previously reported by Nicolaides et al., 1996**[1]**)**			**Family 2**				**Family 3**		
Subject	II-1	III-1^a^	III-2	I-2	II-1	II-2^a^	II-3	I-1^a^	II-1	II-2
Current age	49 yr	22 yr	20 yr	43 yr	15 yr	14 yr	10 yr	40 yr	15 yr	11 yr
Episodes of ataxic encephalopathy and/or weakness										
Age of onset	18 m	16 m	9 m	6 m	9 m	5 yr	18 m	3 yr	1 yr	3 yr
Number of episodes	1	3	1	2	3	1	1	3	3	2
Episodes triggered by febrile illness	+	+	+	+	+	+	+	+	+	+
Age of last episode	18 m	4 yr	9 m	4 yr	7 yr	5 yr	18 m	25 yr	18 m	5 yr
Abnormal eye movements	-	-	-	+	+	-	+	+	+	-
Dysphagia	-	-	-	-	+	-	-	+^b^	-	-
Seizures	-	-	-	-	+^c^	-	-	-	-	-
Symptoms present at most recent examination										
Age at most recent exam	49 yr	22 yr	20 yr	42 yr	14 yr	13 yr	9 yr	39 yr	14 yr	10 yr
Cerebellar ataxia	+	+	+	+	+	+	+	+	+	+
Areflexia	+	+	+	+	+	+	+	+	+	+
Pes cavus	+	+	-	+	-	-	-	-	-	-
Optic atrophy	+	+	+	+	+	+	+	+	+	+
Sensorineural hearing loss	+	+	+	+	+	+	+	+	+	+
Dystonia	-	-	-	+^d^	-	-	-	-	-	-
Urinary symptoms	-	-	-	-	-	-	-	+^e^	-	-
Autonomic dysfunction	-	-	-	-	-	-	-	-	-	-
Cognitive dysfunction	-	-	-	-	+^f^	-	-	-	-	-
Autistic traits	-	-	-	-	-	-	-	-	+^g^	+^g^
Cardiac arrhythmia	-	-	-	+^h^	-	-	-	-	-	-

*Abbreviations* used: m, months; yr, years; +, present; -, absent or not noted.

^a^Proband.

^b^Mild persistent dysphagia was noted by age 32 years.

^c^Brief focal seizures (unilateral arm jerking) developed in association with first episode of fever and ataxic encephalopathy.

^d^Patient developed cervical dystonia responsive to onabotulinumtoxinA injections at age 32 years.

^e^Urinary urgency and frequency were noted by age 32 years.

^f^Patient was assessed at age 10 years for school difficulties and found to have low average to average IQ and reduced attention skills.

^g^Siblings II-1 and II-2 of family 3 were noted to have repetitive behaviours and social difficulties at age 10 and 4 years, respectively.

^h^Patient was diagnosed with Wolff-Parkinson-White syndrome at age 24 years.

### Family 1

Following normal development, the male proband (Figure 1A, Family 1 III-1) of this Caucasian United Kingdom family experienced three episodes of lethargy, hypotonia, and ataxia during acute febrile illnesses, beginning at age 16 months. The most severe episode occurred at age 4.5 years, when he was comatose for a week. Neurologic sequelae at 6 years of age included markedly ataxic gait, poor hand coordination, truncal hypotonia, nystagmus, dysarthria, optic atrophy with absent visual evoked potentials and moderate bilateral sensorineural deafness; the latter two features had developed at age 3 years.

On re-examination at age 22 years, his phenotype was slightly more severe than that described at age 6 years. He had suffered no acute episodes since age 4.5 years but had slow progression of all symptoms since that time. His balance remained poor, and, although he walked unaided, he could not walk on uneven surfaces. He read with the assistance of an electronic magnifier and wore hearing aids. His cognition was normal, and he was studying at university.

His 49-year old mother (Figure 1A, Family 1 II-1) was well until the age of 18 months when she developed acute ataxia during a febrile illness. She experienced no further acute episodes but has shown a progressive course with visual impairment, deafness and loss of balance. At age 31 years she had profound bilateral sensorineural hearing loss, bilateral optic atrophy with horizontal nystagmus, mild cerebellar ataxia, pes cavus, and absent deep tendon reflexes. Since then, her walking has progressively deteriorated: she cannot walk on uneven surfaces and has required a walking stick for the past 2 years. She is now registered blind and requires hearing aids for profound bilateral hearing loss. Her visual evoked potentials and brainstem auditory evoked potentials are absent. Electromyogram showed motor unit loss and innervation changes.

The proband’s 20-year old sister (Figure 1A, Family 1 III-2) presented with an ataxic episode with marked hypotonia, nystagmus and areflexia at age 9 months during a non-specific febrile illness. She recovered but was noted to have optic atrophy shortly after her acute neurological symptoms resolved. She has not had any subsequent ataxic episodes. She has developed sensorineural deafness, but the severity is less than that of her 22-year old brother. She can walk on uneven surfaces but tends to fall when running. Although registered as partially sighted, she does not use any visual aids. She has hearing aids for mild-moderate hearing loss. She is also studying at university.

The proband’s mother’s parents (Figure 1A, Family 1 I-1 and I-2) both have normal neurological examinations. There are two maternal sibs who were re-evaluated recently, and are both unaffected at ages 50 and 46 years, respectively.

### Family 2

The proband of Family 2 (Figure 1A, Family 2 II-2) is now a 14 year-old girl who was well until she developed ataxic encephalopathy with a febrile illness at age 5 years. She had a reduced level of consciousness for the first two weeks and slow recovery afterwards with dysarthria, dysphagia, dysmetria and truncal and gait ataxia. She did not fully recover and was unable to walk without support for one year following the episode. She has had no further episodes, but progressive optic atrophy and sensorineural hearing loss developed, and 9 years later she wears hearing aids for bilateral moderate-to-severe upsloping sensorineural hearing loss. She has bilateral optic atrophy, horizontal end-gaze nystagmus, and 6/46 visual acuity. The patient has mild dysarthria, and, although her gait has deteriorated and she is areflexic, she is still able to walk unaided. She has had mild left ventricular enlargement on cardiac evaluation since early childhood. Her cognition was assessed to be low average to average at age 10 years, and she has had attention and school difficulties.

Her two siblings and father show similar features but their clinical courses have been milder. The proband’s older brother (Figure 1A, Family 2 II-1), who is now 15 years old, presented at 9 months of age during a febrile illness. He had two further episodes of ataxic encephalopathy with febrile illnesses at 2.5 and 7 years of age; transient limitation of abduction or adduction in one or both eyes was also noted during these episodes. He had mild residual balance difficulties following the episodes, but his cerebellar ataxia is currently minimal, with only mild difficulties with tandem gait and standing on one foot. He is areflexic but does not have pes cavus. Sensorineural hearing loss was identified at age 7 years, but his subsequent audiological assessments have been stable, with a mild upsloping sensorineural hearing loss bilaterally. He uses an FM amplification device. He has bilateral optic disc atrophy and horizontal nystagmus, with visual acuity of 6/15 in the right eye and 6/18 in the left eye. He has no academic difficulties. He has mild left ventricular enlargement of the heart that has been stable since early childhood.

The proband’s younger brother (Figure 1A, Family 2 II-3), now aged 10 years, presented at 18 months of age with an acute ataxic episode triggered by a febrile illness. He has had no subsequent acute episodes but has mild residual balance difficulties, minimal cerebellar ataxia, and absent reflexes. He had eye movement difficulties during his acute episode, with a residual left intermittent esotropia that improved with patching. He also has bilateral optic disc atrophy and horizontal nystagmus, with visual acuity of 6/15 bilaterally. He developed a moderate upsloping bilateral sensorineural hearing loss and now wears hearing aids. He has no academic problems.

The father (Figure 1A, Family 2 I-2), who is of French Canadian descent, is currently 43 years of age. When he was 6 months old, he developed fever, generalized hypotonia and weakness, areflexia, vertical nystagmus, and limited abduction of both eyes. He recovered but had a similar episode with fever at age 4 years. Optic disc atrophy and sensorineural hearing loss were also identified at that time. He now has profound sensorineural hearing loss and has had a cochlear implant. He is legally blind. He walks unaided but has mild dysmetria and ataxic gait, areflexia and pes cavus. He was treated for Wolff-Parkinson-White syndrome at age 24 years. He developed cervical dystonia with dystonic tremor at age 32 years and has benefited from onabotulinumtoxinA injections since 38 years of age. Sural nerve biopsy performed at age 34 years revealed findings consistent with a mild-moderate axonal neuropathy.

### Family 3

The proband of Family 3 (Figure 1A, Family 3 I-1), who lives in the United Kingdom and is of Caucasian descent, is a 40-year old woman who presented at age 3 years with a febrile illness and associated weakness and ataxia, which resolved after 4 months. At age 11 years, she had another acute episode, during which she became comatose and was diagnosed with encephalitis. She made a full intellectual recovery but was left with poor vision in association with optic atrophy, severe bilateral sensorineural hearing loss and ataxia. After another febrile illness at age 25 years, she developed generalized weakness and worsening of her hearing, vision and ataxia. Clinical re-evaluation at age 27 years showed optic atrophy with impaired vision (acuity 6/60 in the left eye and 6/36 in the right eye) with pendular nystagmus in all directions of gaze and superimposed square wave jerks. She had severe bilateral sensorineural hearing loss, mild dysarthria, moderate ataxia and absent deep tendon reflexes but no pes cavus. Her cognition was normal. The neurological findings were unchanged at 32 years of age, but she reported swallowing difficulties owing to slow movement of her lips and tongue, and increased urinary urgency and frequency, which responded well to treatment with oxybutynin. She had abnormal visual evoked potentials bilaterally, but her electroretinogram was normal.

The proband’s mother died at age 50 years of “heart problems” but was otherwise said to have enjoyed good health; autopsy was not performed. The proband’s father has no signs of neurologic or systemic disease.

The proband’s 15-year old daughter (Figure 1A, Family 3 II-1) was well until the age of 1 year when she presented with a febrile episode associated with generalized weakness, floppiness and areflexia. She made a full recovery after 5 weeks. She developed similar, but milder, problems following another febrile illness at the age of 18 months. At age 3 years she was found to have increased latencies of her visual evoked potentials and brain-stem auditory evoked potentials. Bilateral mild sensorineural hearing loss was diagnosed at 7 years of age, and optic disc pallor was noted at age 7.5 years. At age 10 years, she had an upper respiratory infection and developed acute onset of strabismus, which took several weeks to resolve. Although her academic progress was reported to be normal, repetitive behaviors and social difficulties resulted in a diagnosis of autism spectrum disorder at age 10.5 years.

The proband's son (Figure 1A, Family 3 II-2), who is now 11 years old, developed profound weakness, hypotonia and areflexia during a febrile illness at age 3 years. He recovered slowly over 6 months, but his brain stem auditory evoked potentials were abnormal and by age 3.5 years he had optic disc pallor and visual acuity of 6/12 in both eyes. He had an ataxic gait and was areflexic. At age 5 years he had another acute febrile episode with weakness, hypotonia and altered sensorium that resolved after 3 days. Bilateral mild low frequency sensorineural hearing loss was diagnosed at age 5.8 years and has been stable since then. He has also been diagnosed with autism spectrum disorder.

### Genetic studies

We used whole exome sequencing to identify heterozygous variants that were present in the same gene in the probands of both Family 1 and Family 2, were rare in the normal population, and were predicted to cause non-synonymous changes in protein-coding regions or to interfere with splicing. The results of this analysis are summarized in Table 2. Candidate variants were validated using PCR and standard Sanger sequencing in all seven affected and four unaffected members of CAPOS Families 1 and 2.

**Table 2:** Summary statistics of whole exome sequencing in two unrelated patients with CAPOS syndrome

	**Family 1, subject III-1**	**Family 2, subject II-2**
Total reads	108,841,666	104,389,738
Chastity-passed reads	103,868,186	102,337,954
Reads aligned with mapping quality ≥10	92,611,315	91,501,102
Average exome read depth^a^	69x	65x
Non-synonymous single nucleotide variants	10,911	11,254
Splice-site single nucleotide variants	517	524
Coding insertions/deletions^b^	805/416	810/501
Non-silent variants^c^ not in dbSNP 129 or 130	2,259	2,209
Novel^d^ heterozygous, autosomal variants	390	224
Genes with novel *non-identical* heterozygous variants in both probands	9^e^	
Genes with novel *identical* heterozygous variants in both probands	3^f^	
Variant segregating in all 10 affected family members tested in three CAPOS families	*ATP1A3*	
	chr19:47,166,267C > T (hg18)^g^	
	c.2452G > A^h^	
	p.Glu818Lys	

^a^Average read depth of exons annotated using Ensembl 54 (including potential PCR duplicates) calculated as (sum of the number of reads aligned per site for all exonic sites) / (total number of exonic sites).

^b^Supported by ≥7 reads.

^c^Includes non-synonymous single nucleotide variants, splice-site single nucleotide variants (within 2 bases of exon boundaries), and small, coding insertions and deletions as annotated using Ensembl 54 gene models.

^d^Not previously reported in dbSNP129 or 130, 1000 Genomes Project, or 1834 non-cancer genomes collected in Canada’s Michael Smith Genome Sciences Centre’s local database.

^e^Non-identical variants of 6 of these genes were called with sufficient quality in both probands to warrant confirmation by Sanger sequencing. Variants of the following genes were tested but did not segregate with the disease in either family: *WDR26, C12orf56, LMO7,* and *DNAH17*. A variant of *SIGLEC1* segregated as expected in Family 2, but the variant found in Family 1 did not segregate as expected. A variant of *MUC16* segregated as expected in Family 1, but neither of two different variants of this gene found in Family 2 segregated as expected.

^f^Only one of these variants (c.2452G > A of *ATP1A3*) was called with sufficient quality in both probands to warrant confirmation by Sanger sequencing*.* This variant showed the expected segregation pattern with the disease in both families.

^g^Location in hg19 is chr19:42,474,427C > T.

^h^Annotation is on Ensembl transcript ENST00000302102, version 5.

Only one novel heterozygous missense variant was demonstrated in all affected members of both families and absent in the two unaffected spouses tested. This variant, which was the same in both families (Additional file 2: Figure S1), occurred at position 47,166,267 (C > T) of chromosome 19 (NCBI36/HG18) and corresponds to c.2452G > A (p.(Glu818Lys)) (Ensembl transcript ENST00000302102, version 5)[9] in the *ATP1A3* (sodium/potassium-transporting ATPase subunit α_3_) gene (OMIM 182350) encoded on the opposite strand. We then analyzed *ATP1A3* by targeted Sanger sequencing in Family 3 and demonstrated the same c.2452G > A mutation in all three affected individuals. Figure 1B shows the *ATP1A3* Sanger sequencing results for Families 1–3.

We found the same *ATP1A3* c.2452G > A mutation in all three CAPOS families studied. This observation raised the possibility that the mutation might exhibit a common origin through unaffected carrier antecedents. However, the *ATP1A3* c.2452G > A mutation in the affected mother (II-1) in Family 1 was not inherited from either of her unaffected parents (Figure 1B, Family 1 I-1 or I-2) and, therefore, must have arisen *de novo.* (Non-paternity was excluded by testing all six Family 1 members shown in Figure 1A with Illumina OMNIExpress whole genome genotyping arrays)(Illumina, Inc., San Diego, Calfiornia, USA).

The parents of the affected adults in Families 2 and 3 were not available for mutation testing, so we performed genome-wide SNP genotyping on Illumina OMNIExpress arrays using standard protocols to determine the haplotype on which the mutation arose. We found that both families shared a region of more than 2 Mb containing 35 informative SNPs surrounding the mutation (Additional file 3: Figure S2). Although new mutations are most likely in all three families, we cannot rule out the possibility of a remote relationship between Families 2 and 3 with a common ancestral *ATP1A3* c.2452G > A mutation and incomplete penetrance.

The heterozygous *ATP1A3* c.2452G > A variant found in all 10 affected individuals in these three CAPOS families was not observed in more than 1834 unaffected individuals who had undergone whole exome or whole genome sequencing.

## Discussion

*ATP1A3* encodes the catalytic α_3_ subunit of Na^+^/K^+^ ATPase, an integral membrane protein responsible for establishing and maintaining electrochemical gradients across the plasma membrane. The c.2452G > A mutation substitutes a positively-charged lysine for a negatively-charged glutamate in the C-terminus cation transporting domain of the Na^+^/K^+^ ATPase α_3_ protein. SIFT [10] predicts this change to be damaging with high confidence (score = 0), and Mutation Taster [11] predicts it to be disease-causing (probability = 1.00). PhyloP [12] indicates that the affected nucleotide is highly conserved (score = 2.17), and this is also apparent by inspection of the amino acid sequence in the altered region of the protein (Figure 1C).

Na^+^/K^+^ ATPase uses adenosine triphosphate (ATP) to pump Na^+^ ions out of cells and K^+^ ions into cells [13,14]. These gradients are involved in regulating neurotransmitter reuptake and the electrical excitability of nerve and muscle. In addition, Na^+^/K^+^ ATPase plays a key role in several signal transduction pathways. We have not studied the effect of the c.2452G > A mutation on Na^+^/K^+^ ATPase function, but our observation of exactly the same missense change in association with the same, extremely rare phenotype as a result of at least two, and probably three, separate mutational events is consistent with a gain of function. Studies of other characteristic phenotypes that are caused by identical recurrent mutations of other genes have often shown that the responsible mutations produce a gain of function e.g., [15-17].

Although the c.2452G > A mutation has not been reported before, different mutations of *ATP1A3* are known to cause two other autosomal dominant neurological diseases: rapid-onset dystonia-parkinsonism [18-20] (DYT12; OMIM: 128235) and alternating hemiplegia of childhood [21,22] (AHC; OMIM: 614820). These two disorders have somewhat overlapping clinical features but are generally considered to be distinct [22-24]. Moreover, the *ATP1A3* mutations reported in the two conditions all differ, and most alternating hemiplegia of childhood mutations occur *de novo*, while rapid-onset dystonia-parkinsonism mutations are more often inherited [21-24]. This genotype-phenotype correlation also extends to CAPOS syndrome in that the mutation we observed has not been seen in either of the other conditions associated with *ATP1A3* mutations, and the clinical features of CAPOS syndrome, although somewhat overlapping, are distinct from those of rapid-onset dystonia-parkinsonism or alternating hemiplegia of childhood. In addition, all of the *ATP1A3* mutations associated with DYT12 or AHC that have been assessed functionally produce loss of function [18,20,21], while the recurrent c.2452G > A variant found in the three CAPOS syndrome families presented here has characteristics of a gain-of-function mutation, as discussed above.

Table 3 provides an overview of the phenotypic similarities and differences between the three conditions. CAPOS syndrome, DYT12 and AHC all can be inherited as autosomal dominant traits, and all three are characterized by variable expressivity. All may exhibit acute onset of neurological symptoms in childhood in association with a febrile illness, but the predominant neurological manifestations differ – ataxic encephalopathy in CAPOS syndrome, dystonia/parkinsonism in DYT12, and transient episodes of hemiplegia and other symptoms including dystonia in AHC.

**Table 3:** **Comparison of clinical features in alternating hemiplegia of childhood (AHC), rapid-onset dystonia-parkinsonism (DYT12) and CAPOS syndrome (adapted and modified from Rosewich et al.**[22]**)**

	**Alternating hemiplegia of childhood**	**Rapid-onset dystonia-parkinsonism**	**CAPOS syndrome**
**Usual age of onset**	0 – 18 m	4 – 55 yr	6 m – 5 yr
**Onset trigger**			
Emotional stress	?^a^	+	?^a^
Exercise	?^a^	+	?^a^
Hypo/Hyperthermia	+	+	+^b^
Bathing	+	Not reported	-
Alcohol	?^a^	+	?^a^
**Neurological symptoms**			
Ataxic encephalopathy episodes	-	-	**+**
Hemiplegic episodes	+	-	-
Quadriplegic or paretic episodes	+	-	+
Dystonia	+	+	−/+
Dysarthria	+	+	−/+
Drooling	+	+	−/+
Reduced facial expression	−/+	+	-
Mutism	−/+	+	-
Rostrocaudal gradient	+	+	-
Ataxic gait	−/+	+	+
Bradykinesia	−/+	+	-
Seizures	−/+	−/+	−/+
Choreoathetosis	+	-	-
Abnormal eye movements during episodes	+	−/+	−/+
Areflexia	-	-	+
Pes cavus	-	-	−/+
Optic atrophy/visual loss	-	-	**+**
Sensorineural hearing loss	-	-	**+**
Developmental delay or intellectual disability	+	−/+	−/+
**Clinical course**			
Abrupt onset	+	+	+
Polyphasic with slow progression of non-paroxysmal symptoms	+	-	−/+
Mono- or biphasic with slow progression of neurological symptoms	-	+	−/+

*Abbreviations used:* m, months; yr, years; +, feature usually occurs; +/−, feature may occur; -, feature not described.

^a^Cannot assess in infancy or early childhood.

^b^Hyperthermia only.

In contrast to AHC and DYT12, in which the dystonic symptoms are often asymmetric and progress in a rostrocaudal gradient, dystonia is an uncommon feature in CAPOS syndrome. The clinical features that progress in this disorder are more generalized and symmetric; they include progressive gait ataxia and loss of vision and hearing. Bulbar symptoms, which typically occur in AHC and DYT12, are uncommon in CAPOS syndrome.

Optic atrophy and sensorineural hearing loss have not been reported in DYT12 or AHC but are frequent features of CAPOS syndrome. These symptoms in our patients are slowly progressive over time. The recent demonstration that the Na^+^/K^+^-ATPase α_3_ subunit plays a critical role in anchoring retinoschisin, the protein involved in X-linked juvenile retinoschisis, to photoreceptor and bipolar cells of the retina in a mouse model is compatible with involvement of *ATP1A3* in visual function [25]. The role of the Na^+^/K^+^-ATPase α_3_ subunit in hearing is unknown, but *ATP1A3* is abundantly expressed in membranes of spiral ganglion somata, type I afferent terminals contacting inner hair cells and medial efferent terminals contacting the outer hair cells of the cochlea [26].

Unaffected carriers have been reported for other *ATP1A3* missense mutations in rapid-onset dystonia-parkinsonism [18,19,27], but there is no evidence for incomplete penetrance in CAPOS syndrome. We demonstrated a *de novo* origin for the *ATP1A3* c.2452G > A mutation in Family 1 (Figure 1B), and the clinical and family histories are most compatible with separate, recurrent *de novo* mutations in Families 2 and 3 as well.

We conclude that a heterozygous c.2452G > A mutation in *ATP1A3* causes CAPOS syndrome in ten affected individuals in three different families. Clinically, CAPOS syndrome is characterized by acute onset of ataxic encephalopathy with febrile illness in childhood, partial recovery and subsequent slow progression. Testing for *ATP1A3* mutations should be considered in other patients with features of CAPOS syndrome or with other paroxysmal and progressive forms of early-onset dystonia, weakness or ataxia.

## Competing interests

The authors declare that they have no competing interests.

## Authors’ contributions

The study was conceived and designed by MKD, CDMvK, SA, SJMJ, and JMF. Data were acquired by MKD, CDMvK, CJDR, SA, GH, MS, AF, and SJMJ. Data were analysed and interpreted by MKD, CDMvK, CJDR, YS, SHZ, CS, GH, MS, AF, SJMJ, and JMF. The study was supervised by SJMJ and JMF. The manuscript was prepared by MKD and CDMvK and critically revised for important intellectual content by JMF. All authors approved the manuscript.

## Supplementary Material

Additional file 1: Table S1 Investigations performed on 10 patients from three families with CAPOS syndrome. Click here for file

Additional file 2: Figure S1 Visualization of read alignments supporting the *ATP1A3* mutation in the libraries from each of the two probands. Upper panel: Family 1 subject III-1. Lower panel: Family 2 subject II-2. Read alignments to hg18 stored in BAM files were manually examined, and the alignment image was exported using Integrated Genome Viewer [28,29]. The heterozygous C > T mutation at chromosome 19:47,166,267 was corroborated by 22 out of 41 reads in Family 1 subject III-I and by 34 out of 59 reads in Family 2 subject II-2. Click here for file

Additional file 3: Figure S2 Haplotyping results in Families 2 and 3 in the region flanking the *ATP1A3* mutation. Click here for file
