# Supplementary material for: A novel recurrent mutation in ATP1A3 causes CAPOS syndrome
Source: Orphanet J Rare Dis. 2014 Jan 28;9:15. doi: 10.1186/1750-1172-9-15 (PMC3937150; doi:10.1186/1750-1172-9-15)
Supplement: Additional file 1: Table S1 — Investigations performed on 10 patients from three families with CAPOS syndrome. [file 1750-1172-9-15-S1.docx]

**Legends for Supplementary Figures**

**Supplementary Figure S1. Visualization of read alignments supporting the *ATP1A3* mutation in the libraries from each of the two probands.** Upper panel: Family 1 subject III-1. Lower panel: Family 2 subject II-2. Read alignments to hg18 stored in BAM files were manually examined, and the alignment image was exported using Integrated Genome Viewer [28, 29]. The heterozygous C>T mutation at chromosome 19:47,166,267 was corroborated by 22 out of 41 reads in Family 1 subject III-I and by 34 out of 59 reads in Family 2 subject II-2.

**Supplementary Figure S2. Haplotyping results in Families 2 and 3 in the region flanking the *ATP1A3* mutation.**

**Supplementary Table S1**. **Investigations performed on 10 patients from three families with CAPOS syndrome.**

| **Subject** | **Family 1**  (Previously reported by Nicolaides et al. [1]) | | | **Family 2** | | | | **Family 3** | | |
| --- | --- | --- | --- | --- | --- | --- | --- | --- | --- | --- |
|  | II-1 | III-1^a^ | III-2 | I-2 | II-1 | II-2^a^ | II-3 | I-1^a^ | II-1 | II-2 |
| Hematology studies | ND | CBC, vacuolated lympho-cytes, acantho-cytes, immuno-globulins, and IgG subclasses: all normal | ND | CBC: normal | CBC: normal | CBC and ferritin: normal | CBC and immuno-globulins: normal |  | CBC, ESR, and vacuolated lympho-cytes (age 1 yr): normal | CBC and clotting screen (age 3 yr): normal |
| Blood chemistry | ND | ESR, AST, ALT, urate, ammonia, chole-sterol, tri-glycerides, apo-proteins A and B, vitamins A, B12, E, and folate, and AFP: all normal | ND | Ammonia, lactate, AFP, CPK, chole-sterol, creatine, and vitamin E: all normal | Lactate, ammonia, and CPK: normal | Lactate, CPK, and glucose: normal | Lactate, ammonia, vitamin E, CPK, AST, ALT, and lipoprotein electro-phoresis: all normal | Urea, electro-lytes, liver function tests, ammonia, lactate, immune-globulins, toxicology screen (age 11 yr): all normal | Ammonia: moderate elevation at 1 yr but normal at 3 yr; Urea, electro-lytes, liver function tests, and CPK: all normal | Urea, electro-lytes, liver function tests, ammonia, calcium, magnes-ium: all normal |
| Endocrine studies | ND | Thyroid function and auto-antibodies: normal | ND | TSH | TSH | TSH | TSH |  |  |  |
| Metabolic testing | ND | Plasma and CSF amino acids, VLCFA, phytanic acid, bile acids, copper, cerulo-plasmin, urine amino acids, urine organic acids, urine porphyria screen, and fibroblast PDH and PC activity: all normal | Plasma amino acids, VLCFA, phytanic acid, bile acids, copper, cerulo-plasmin, urine amino acids, urine organic acids, and urine porphyria screen: all normal | Urine organic acids, urine amino acids, VLCFA, and carnitine: all normal | Urine organic acids: normal | CSF lactate and amino acids: normal | Pyruvate, plasma amino acids, urine organic acids, and CSF lactate and amino acids: all normal | Plasma amino acids, plasma lactate and pyruvate, urine organic and amino acids (age 11 yr): all normal;  VLCFA and ammonia (age 27 yr): normal | Plasma amino acids, lactate, AFP, white cell lysosomal enzymes, carnitine, VLCFA, urine organic acids, amino acids, orotic acid and porphyrins, and fibroblast  fatty acid beta- oxidation studies (age 3 yr): all normal | Plasma lactate |
| Mutation testing for nuclear genes | ND | *SCA1:* normal | ND | *OPA-1, FXA, SCA2, SCA3, SCA6, SCA7, POLG1, TWINKLE, ANT1,* and *TK2*: all normal |  | *OPA-1:* normal |  |  |  |  |
| Testing for mitochondrial mutations | ND | *MELAS, MERFF, NARP,* and *LHON*: all normal | ND | Long PCR for mito-chondrial DNA deletions and mutations: normal |  |  |  | Whole mitoch-ondrial genome analysis: no pathogenic variant found |  |  |
| Chromosomal microarray analysis | ND | ND | ND | Affymetrix 6.0: normal |  | Affymetrix 6.0: normal |  |  |  |  |
| Neuroimaging | MRI (age 31 yr): normal | CT (age 2 yrs) and MRI (age 4.5 yrs): normal | MRI (age 2 yr): normal | MRI (49yr): normal |  | MRI and MRS (11 yr): normal | MRI (2 yr): normal | CT (age 11 yr) and MRI (age 27 yr): normal | MRI (age 1 and 7.5 yr): normal | MRI (age 3 yr): normal |
| Neurophysiology studies | VEP and BSAEP: absent; EMG: motor unit loss and innervation changes; SSEP and ERG: normal | VEP: absent;   EEG, ERG, NCS, and EMG: all normal (ages 2 and 4.5 yrs) | ND | EEG (age 1yr), EMG, and NCS (age 44 yr): all normal | EEG (age 7 yr): normal | ERG (age 8 yr), EEG, EMG and NCS (age 11 yr): all normal | ERG (age 2 yr): normal | Pattern VEP: absent bilaterally; flash VEP: reduced amplitudes bilaterally (age 27 yr); EEG (age 3 yr), EMG, NCS, and ERG: all normal | VEP and BSAEP: increased latencies (age 3 yr); EEG and ERG: normal | BSAEP: absent waves II and V bilaterally at 90 dB (age 3 yr) |
| Cerebrospinal fluid analysis | ND | Protein, lactate, glucose, cell count, bacterial culture, and viral antibody titres: all normal | ND | Cell count, glucose, and protein: normal | Cell count, glucose, and protein: normal | Cell count, glucose, protein, lactate, and amino acids: all normal | Cell count, glucose, protein, lactate, and amino acids: all normal | Cell count, glucose, and protein: all normal (age 3 yr) | Cytology, biochem-istry and micro-biology: all normal (age 1 yr) | Cytology, protein, glucose, lactate, glycine, and micro-biology: all normal (age 3 yr) |
| Mitochondrial respiratory chain enzyme analysis | ND | Complex I, II, III and IV: all normal | ND | Complex I, II, III and IV: all normal | ND | ND | ND | Complex I, II, III and IV: all normal | Complex I, II, III and IV: all normal (age 3 yr) | ND |
| Muscle histology | ND | Normal | ND | Moderate non-specific atrophy of type II fibres and large and dysmor-phic mito-chondria on EM (age 34 yr); Non-specific changes, not in support of mito-chondrial disorder (age 38 yr) | ND | ND | ND | Mildly increased fibre size, occasional severely atrophic fibres, no ragged-red or COX-negative fibres, mito-chondrial clumping in smooth muscle cells of arterioles in NADH-TR prepar-ations (age 27 yr) | Normal (age 3 yr) | ND |
| Nerve histology | ND | ND | ND | Sural nerve: mild-moderate axonal neuro-pathy (age 34 yr) | ND | ND | ND | ND | ND | ND |
| Cardiac studies | ND | ECG and echocardi-ogram: normal | ND | Wolff-Parkinson-White syndrome (age 24yr) | Slightly large left ventricle | Slightly large left ventricle | ND | ECG: sinus brady-cardia, probably related to treatment with beta-blocker for hyper-tension (age 39 yr) | ND | ND |

Abbreviations used: AFP, alpha-fetoprotein; ALT, alanine aminotransferase; AST, aspartate aminotransferase; BSAEP, brainstem auditory evoked potentials; CBC, complete blood count; COX, cytochrome oxidase; CPK, creatine phosphokinase; CSF, cerebrospinal fluid; CT, computerized tomography; ECG, electrocardiogram; EEG, electroencephalogram; EM, electron microscopy; EMG, electromyogram; ERG, electroretinogram; ESR, erythrocyte sedimentation rate; IgG, immunoglobulin G; MRI, magnetic resonance imaging; NADP-TR, diaphorase; NCS, nerve conduction studies; ND, not done; PC, pyruvate carboxylase; PDH, pyruvate dehydrogenase; SSEP, somatosensory evoked potentials; TSH, thyroid stimulating hormone; VEP, visual evoked potentials; VLCFA, very long chain fatty acids; VUS, variant of unknown significance; yr, years.

^a^ Proband.
