# Supplementary figures and images for: A novel recurrent mutation in ATP1A3 causes CAPOS syndrome
Source: Orphanet J Rare Dis. 2014 Jan 28;9:15. doi: 10.1186/1750-1172-9-15 (PMC3937150; doi:10.1186/1750-1172-9-15)

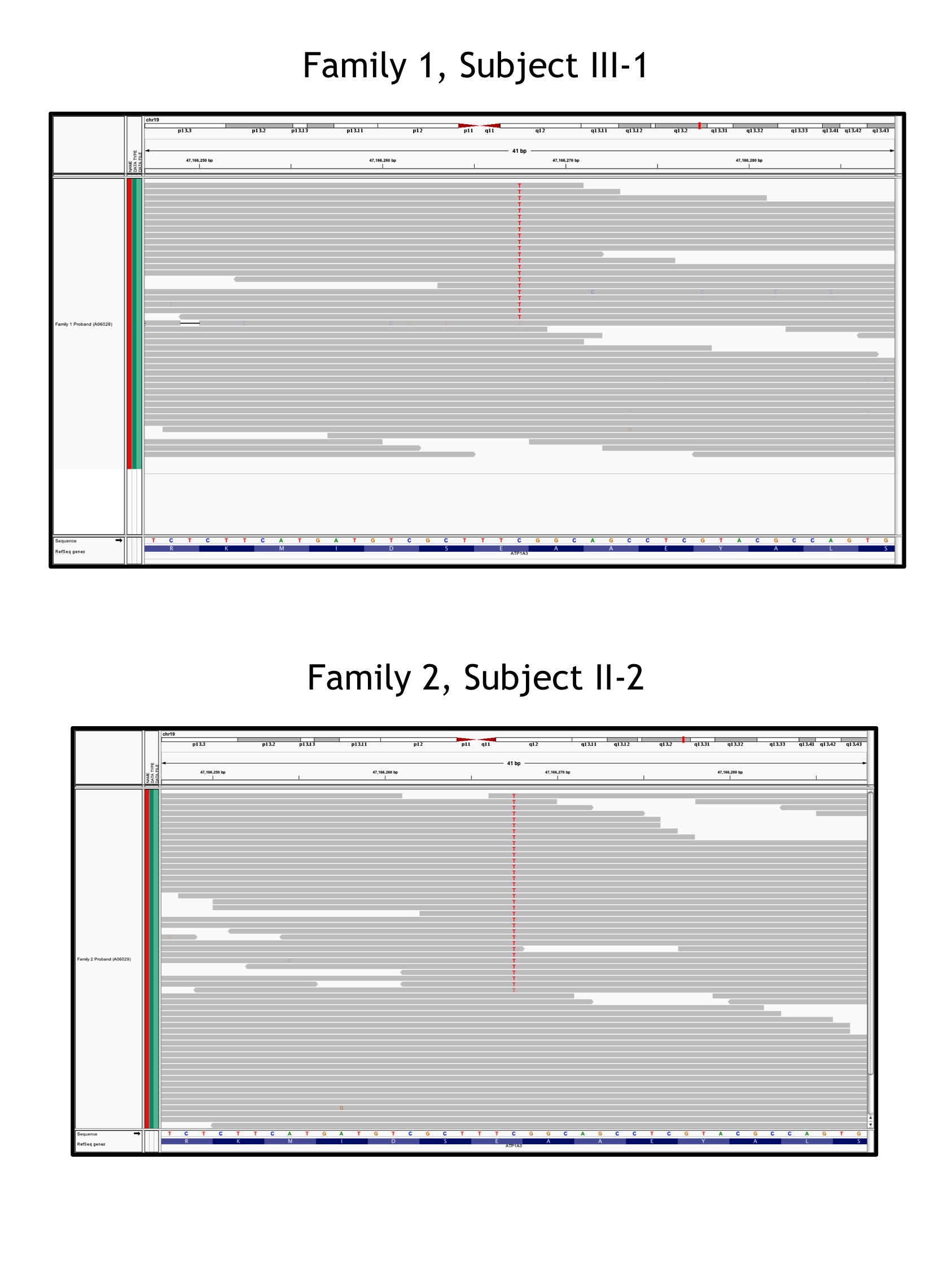

Supplement: Additional file 2: Figure S1 — Visualization of read alignments supporting the ATP1A3 mutation in the libraries from each of the two probands. Upper panel: Family 1 subject III-1. Lower panel: Family 2 subject II-2. Read alignments to hg18 stored in BAM files were manually examined, and the alignment image was exported using Integrated Genome Viewer [28,29]. The heterozygous C > T mutation at chromosome 19:47,166,267 was corroborated by 22 out of 41 reads in Family 1 subject III-I and by 34 out of 59 reads in Family 2 subject II-2. [file 1750-1172-9-15-S2.jpeg]

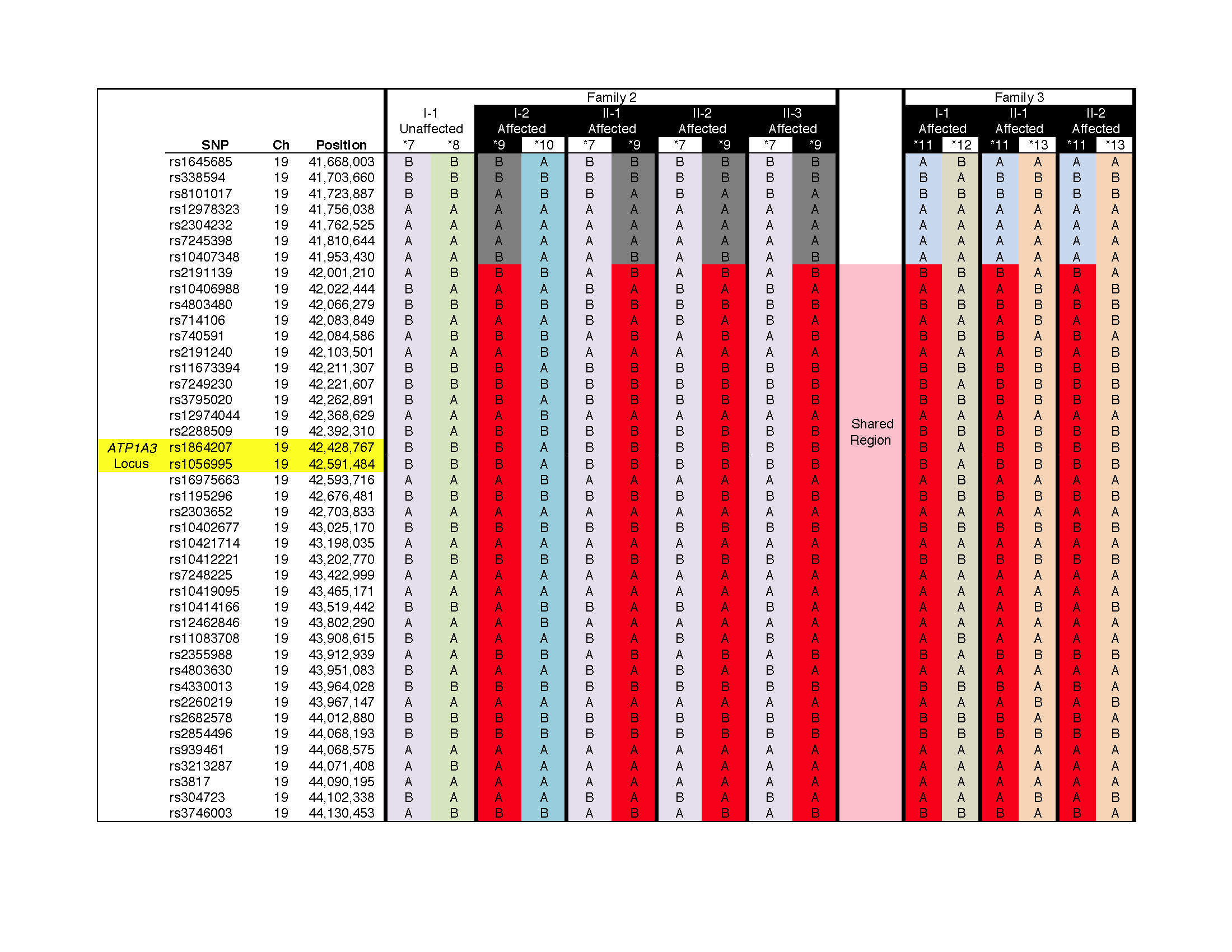

Supplement: Additional file 3: Figure S2 — Haplotyping results in Families 2 and 3 in the region flanking the ATP1A3 mutation. [file 1750-1172-9-15-S3.jpeg]
